# Identification of MarvelD3 as a tight junction-associated transmembrane protein of the occludin family

**DOI:** 10.1186/1471-2121-10-95

**Published:** 2009-12-22

**Authors:** Emily Steed, Nelio TL Rodrigues, Maria S Balda, Karl Matter

**Affiliations:** 1 Department of Cell Biology, UCL Institute of Ophthalmology, University College London, Bath Street, London EC1V 9EL, UK

## Abstract

**Background:**

Tight junctions are an intercellular adhesion complex of epithelial and endothelial cells, and form a paracellular barrier that restricts the diffusion of solutes on the basis of size and charge. Tight junctions are formed by multiprotein complexes containing cytosolic and transmembrane proteins. How these components work together to form functional tight junctions is still not well understood and will require a complete understanding of the molecular composition of the junction.

**Results:**

Here we identify a new transmembrane component of tight junctions: MarvelD3, a four-span transmembrane protein. Its predicted transmembrane helices form a Marvel (MAL and related proteins for vesicle traffic and membrane link) domain, a structural motif originally discovered in proteins involved in membrane apposition and fusion events, such as the tight junction proteins occludin and tricellulin. In mammals, MarvelD3 is expressed as two alternatively spliced isoforms. Both isoforms exhibit a broad tissue distribution and are expressed by different types of epithelial as well as endothelial cells. MarvelD3 co-localises with occludin at tight junctions in intestinal and corneal epithelial cells. RNA interference experiments in Caco-2 cells indicate that normal MarvelD3 expression is not required for the formation of functional tight junctions but depletion results in monolayers with increased transepithelial electrical resistance.

**Conclusions:**

Our data indicate that MarvelD3 is a third member of the tight junction-associated occludin family of transmembrane proteins. Similar to occludin, normal expression of MarvelD3 is not essential for the formation of functional tight junctions. However, MarvelD3 functions as a determinant of epithelial paracellular permeability properties.

## Background

Tight junctions comprise the most apical of the junctional structures in epithelial cells and form a diffusion barrier allowing for the regulated movement of ions and solutes through the paracellular pathway [1]. Paracellular transport is driven by concentration gradients and is size- and ion-selective; however, the molecular mechanisms that permit selective paracellular diffusion are only partially understood. Tight junctions also participate in the establishment and maintenance of cell polarity and in various signalling pathways controlling gene expression, cell differentiation and proliferation. Their ability to perform such an array of functions is largely attributable to the diverse protein complement from which they are composed.

There are two main classes of transmembrane proteins found at the tight junction: the four- and the single-span transmembrane proteins [2-4]. While both classes have been implicated in the adhesive properties of the tight junction, only the four-pass transmembrane proteins â€“ namely claudins, occludin and tricellulin â€“ have so far been directly linked to the barrier properties of the junction. The single-span proteins (e.g., JAMs, Crb3) as well as Bves, a protein with three transmembrane domains, serve different types of regulatory and signalling functions during differentiation, junction assembly, and transmigration of leukocytes [5-9].

Claudins are believed to be the main structural component of the tight junction strands [10,11]. They are thought to form regulated aqueous pores or channels that enable the passive diffusion of charged molecules through the paracellular space [12-14]. Claudin expression and activity are hence thought to be major determinants of paracellular ion conductance. As altered expression of various claudins has been linked to carcinogenesis and cell migration, claudins may also modulate subcellular signalling mechanisms and possess non-junctional functions in the regulation of integrin function [15-24].

A distinct group of tight junction-associated proteins is represented by occludin and tricellulin, both also components of intramembrane strands [25-27]. Based on functional studies in tissue culture cell lines, animal models, as well as inherited human diseases, it seems that occludin and tricellulin possess regulatory roles in junction function, and, at least in the case of occludin, participate in signalling pathways regulated by tight junctions [27-40]. Whereas experiments with tissue culture cells suggest that tricellulin directly contributes to the junctional structure, no such evidence has been reported for occludin [27,39]. Nevertheless, it has recently been demonstrated that occludin depletion results in a redistribution of tricellulin, suggesting that the latter protein may be able to compensate for some functions of the former in occludin knockdown cells [41]. It is thus important to determine whether there are other members of the occludin family at tight junctions.

The four transmembrane helix architecture of both occludin and tricellulin represents a Marvel domain (MAL and related proteins for vesicle traffic and membrane link) [42]. While the prevalence and significance of the Marvel domain is not yet clear, its identification in proteins of the MAL, physin, gyrin and occludin families has lead to putative roles in cholesterol-rich membrane apposition and fusion events to be proposed. Whether all Marvel domain proteins are indeed associated in such membrane apposition events, however, is not clear. The bioinformatics analysis by Sanchez-Pulido and colleagues not only identified Marvel domain-containing proteins of known functions, but also proteins that had previously not been analysed such as Marvel domain-containing protein 2, which was later named tricellulin, and Marvel domain-containing protein 3 (MarvelD3), a protein that has not yet been studied [42].

In this study, we identify MarvelD3, a four-pass transmembrane protein of about 40 kDa, as a novel integral membrane component of the epithelial cell tight junctions. MarvelD3 co-localises with the tight junction protein occludin but not with the adherens junction marker E-cadherin. Functional analysis suggests that depletion of MarvelD3 does not interfere with the formation of functional tight junctions but results in increased transepithelial electrical resistance (TER), suggesting that MarvelD3 is a determinant of paracellular ion permeability.

## Results

### Expression of MarvelD3 in epithelial and endothelial cells

**Figure 1:**
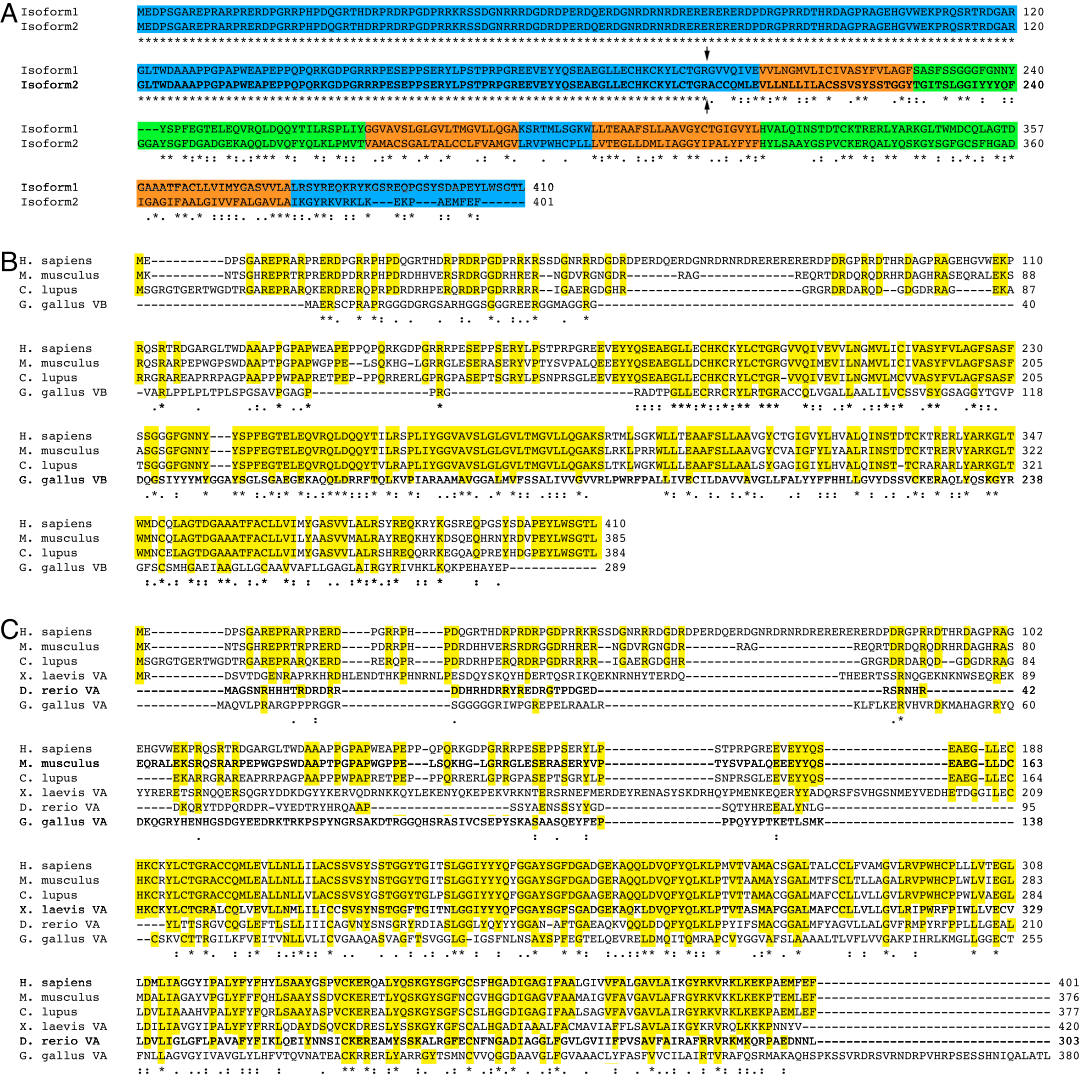
**Analysis of vertebrate MarvelD3 sequences**. (A) Human MarvelD3 isoforms; (B) human, mouse and dog isoform 1 and chicken variant B; and (C) human, mouse and dog isoform 2 and chicken, Xenopus and zebrafish variant A were aligned with ClustalW http://www.ebi.ac.uk/Tools/clustalw2/ using default settings. In panel A, the cytosolic domains are highlighted in blue, the transmembrane domains in orange, and the extracellular domains in green. The splice junction between the N-terminal domain shared by both isoforms and the alternative domains is indicated by two arrows. In panels B and C, the amino acid residues conserved in mammalian MarvelD3 sequences are highlighted in yellow. Conservation is labelled according to ClustalW definitions: identical residues (*); conserved substitutions (:); semi-conserved substitutions (.).

Bioinformatics analysis revealed the existence of two human MarvelD3 isoforms: isoform 1 contains 410 amino acids [Genbank: NM_001017967] and isoform 2 401 amino acids [Genbank: NM_052858] (Fig. 1A). A membrane topology analysis with Phobius http://www.ebi.ac.uk/Tools/phobius/ and TMpred http://www.ch.embnet.org/software/TMPRED_form.html confirmed that both isoforms are predicted to contain four transmembrane domains and to expose their N- and C-terminal domains to the cytosol. The two isoforms represent splice variants and share the predicted N-terminal cytoplasmic domain of 198 amino acids, but differ in their C-terminal halves that contain the transmembrane domains. Both MarvelD3 isoforms are predicted to possess only short C-terminal cytoplasmic domains (30aa isoform1; 18aa isoform 2) with no apparent similarities to the comparatively long cytoplasmic domains of tricellulin and occludin that contain their ZO-1 binding sites [38,43].

Database searches revealed that MarvelD3 is expressed in chicken, Xenopus and various mammalian species, but not in any invertebrates, suggesting that it is expressed by vertebrates only (Fig. 1B and Table 1). Alternatively spliced isoforms were only found in mammalian species. In contrast, the chicken genome contains two distinct MarvelD3 genes, variant A and B, that reside on different chromosomes. Variant A is more similar to mammalian isoform 2. Although variant B is more similar to isoform 1 than 2, the two chicken proteins show a similar degree of conservation with mammalian isoform 1. It thus seems that mammalian isoform 2 and the variant A gene found in birds, fish and amphibians represent the MarvelD3 form common to all vertebrates.

**Table 1:** Alignment scores of MarvelD3 sequences from different vertebrates

	**H. sapiens isoform 1 **NM_001017967	**H. sapiens isoform 2 **NM_052858	**M. musculus isoform 1 **NM_212447	**M. musculus isoform 2 **NM_028584	**C. lupus f. Isoform 1 **XM_848243	**C. lupus f. Isoform 2 **XM_546843	**X. laevis Variant A **BC_068841	**G. gallus Variant A **XM_418989	**G. gallus Variant B **XM_414239
H. sapiens isoform 2 NM_052858	63	100							

M. musculus isoform 1 NM_212447	72	40	100						

M. musculus isoform 2 NM_028584	43	68	61	100					

C. lupus f. Isoform 1 XM_848243	71	39	67	39	100				

C. lupus f. Isoform 2 XM_546843	40	69	38	68	61	100			

X. laevis Variant A BC_068841	25	43	24	42	23	44	100		

G. gallus Variant A XM_418989	30	51	28	48	28	50	44	100	

G. gallus Variant B XM_414239	30	24	30	21	28	22	23	24	100

D. rerio Variant A BC_055662	25	40	26	37	23	40	39	31	25

MarvelD3 sequences were retrieved from Genbank and aligned using ClustalW (see Fig. 1 for a selection of the alignments). Indicated are all Genbank accession numbers. The scores reflect percent identity. For mammals, the two alternatively spliced isoforms were used. Two different MarvelD3 genes, variants A and B, exist in chicken. Only variant A was found in X. laevis and zebrafish.

We first generated an antibody against a peptide of the N-terminal cytoplasmic domain to analyse MarvelD3 expression. Expression was analysed in two epithelial cell lines derived from different types of epithelia: Caco-2, a human colon adenocarcinoma cell line, and an immortalised human corneal epithelial cell line (HCE) [7,44]. Total cell extracts were generated from control cells as well as cells transfected with control siRNAs or a pool of four siRNAs targeting MarvelD3. All four targeted sequences are part of the common exon encoding the N-terminal cytoplasmic domain; hence, mRNAs encoding both isoforms should become degraded.

**Figure 2:**
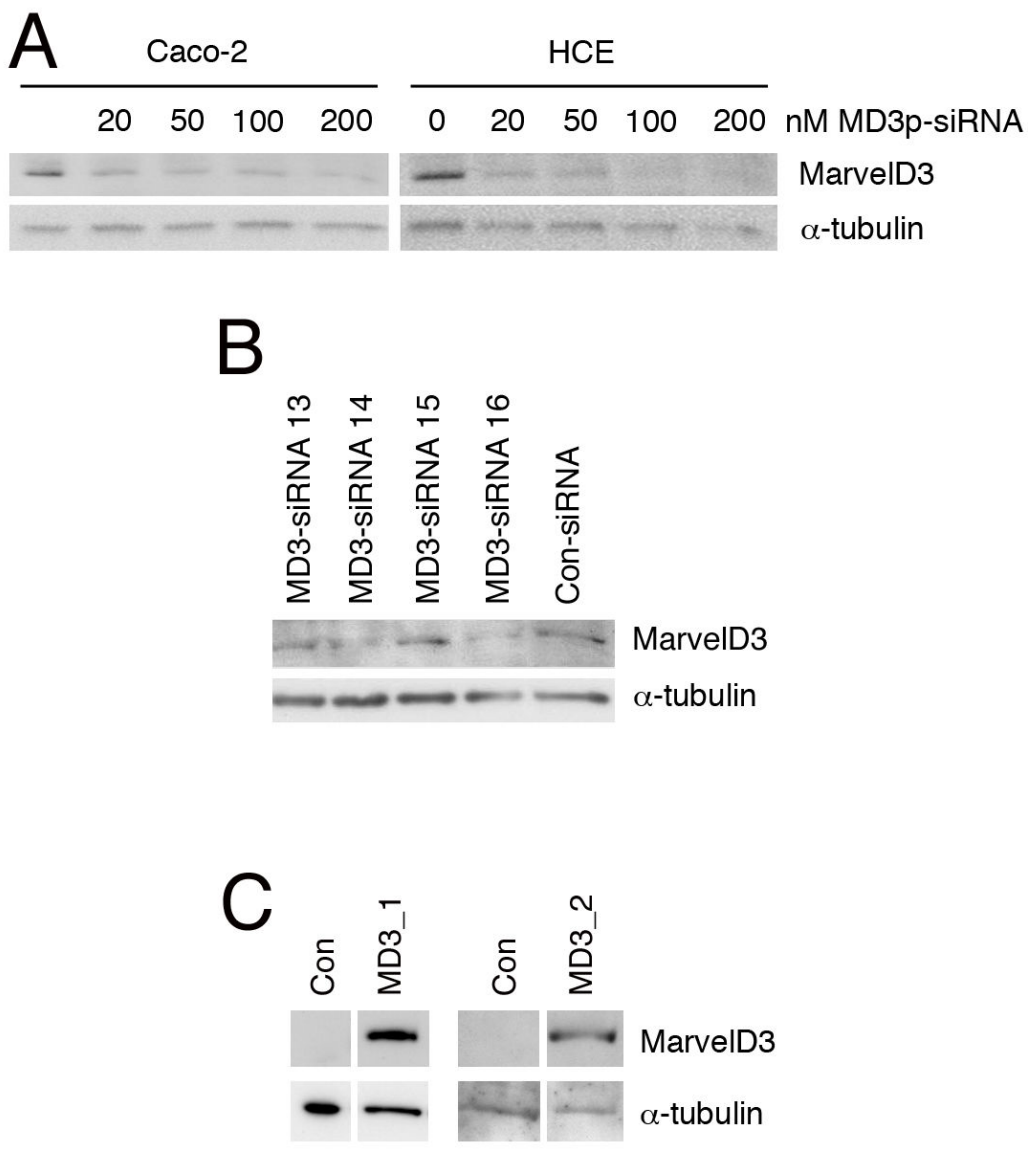
**Expression of MarvelD3 in epithelial cell lines**. (A) Endogenous levels of MarvelD3 proteins in Caco-2 and HCE cells. The two cell lines were transfected with the indicated concentrations of siRNAs, using a pool of the four MarvelD3-directed siRNAs. After cell lysis, expression levels of MarvelD3 and α-tubulin were analysed by immunoblotting. (B) Identification of functional siRNAs. Caco-2 cells were transfected with individual siRNAs targeting MarvelD3 or control siRNAs. Depletion of MarvelD3 was then analysed by immunoblotting. (C) Exogenous expression of MarvelD3 isoforms. Caco-2 cells were transfected with cDNAs encoding either isoform 1 or isoform 2 of MarvelD3. Expression was then analyzed by immunoblotting with anti-MarvelD3 antibody. MD3_1 and MD3_2 constructs were run on separate SDS-PAGE gels and are shown alongside control transfections run on the same gels. Note, endogenous levels of MarvelD3 are only detected at longer exposures than those used to detect transfected proteins.

Fig. 2A shows that the antibody recognised a band of about 40 kD in both cell lines as expected. The band became weaker with increasing concentrations of MarvelD3 siRNA, indicating that the band indeed corresponded to MarvelD3. Deconvolution of the MarvelD3 siRNA pool revealed that siRNAs 14 and 16 were the most effective of the four sequences and were hence used for the subsequent functional analysis (Fig. 2B). Transfection of cDNAs encoding the two isoforms resulted in strong bands of the same molecular weight in immunoblots with the anti-MarvelD3 antibody, further supporting its specificity (Fig. 2C; the bands in control lanes are not visible as all samples were exposed for the same period of time). Further immunoblotting experiments revealed MarvelD3 protein expression in additional cell lines derived from prostate and mammary gland (not shown). These observations indicate that MarvelD3 is expressed in different epithelial cell types.

**Figure 3:**
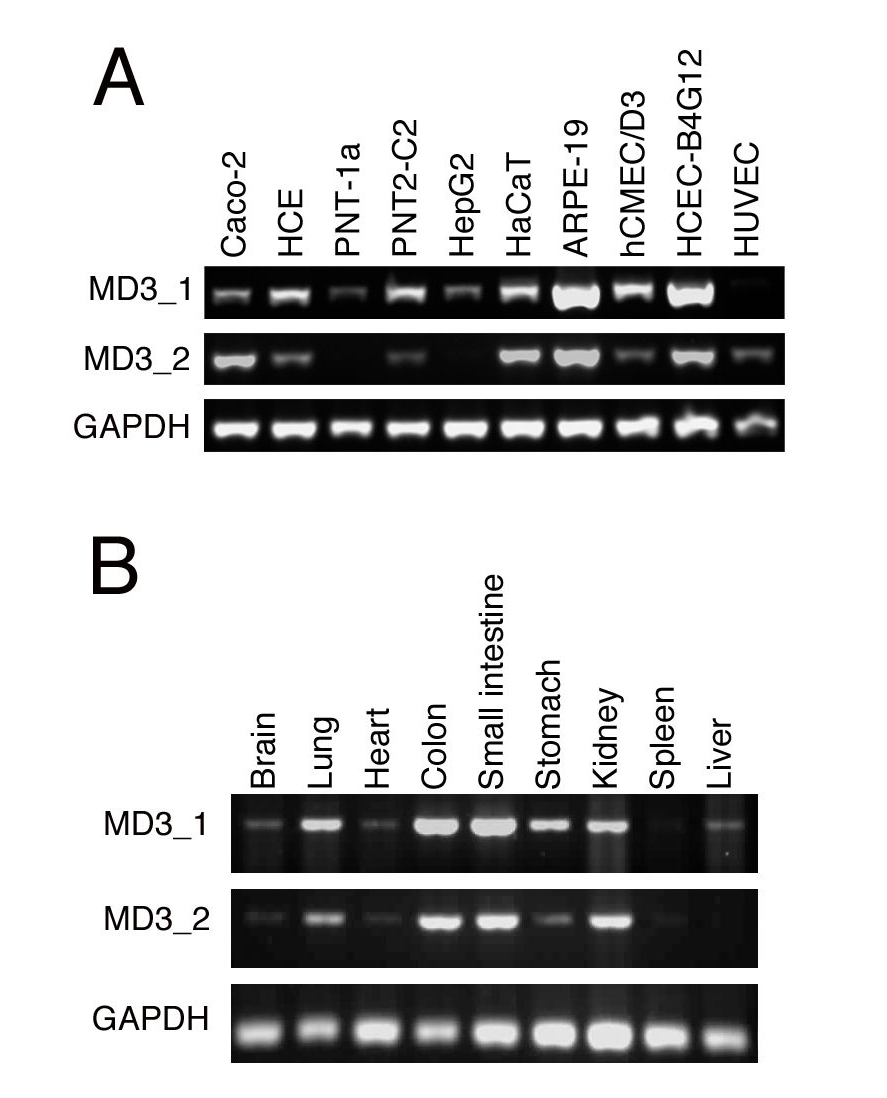
**Expression of MarvelD3 isoforms in different epithelial and endothelial cells**. Reverse transcription PCR was used to analyse the expression of MarvelD3 isoforms in cultured human epithelial and endothelial cells (A) and adult mouse tissues (B). Primers were used to specifically amplify MarvelD3 isoforms or, as a control, GAPDH. In panel A, cell lines and primary cultures derived from the following cell types were used: Caco-2, colon adenocarcinoma cells; HCE, immortalised corneal epithelial cells; PNT-1a and PNT2-C2, immortalised prostate epithelial cells; HepG2, hepatocellular carcinoma cells; HaCaT, spontaneously immortalised skin keratinocytes; ARPE-19, spontaneously immortalised retinal pigment epithelial cells; hCMEC/D3, immortalised brain endothelial cells; HCEC-B4G12, immortalised corneal endothelial cells; HUVEC, umbilical vein endothelial cells. Note, mRNAs for both isoforms are widely expressed by epithelial and endothelial cells.

The MarvelD3 antibody was found to recognise only human MarvelD3 and recognises both isoforms; hence, we used reverse transcription PCR to determine expression of MarvelD3 isoforms in different cultured epithelial and endothelial cell lines, as well as different tissues. Fig. 3A shows that both isoforms are widely expressed by different epithelial and endothelial cells. Similarly, most tested adult mouse tissues expressed both isoforms (Fig 3B). Both MarvelD3 isoforms are thus widely expressed and are found in different types of epithelial and endothelial cells. Nevertheless, apparent differences in isoform expression profiles were detected as, for example, both liver and the hepatocyte-derived cell line HepG2 only expressed isoform 1.

### Localisation of MarvelD3

We next used indirect immunofluorescence microscopy to determine the localisation of MarvelD3 in epithelial cells. As our antibody only recognises the human protein, we used Caco-2 and HCE cells for the localisation experiments as they form well-developed junctional complexes and are derived from two different types of epithelia. Confluent cultures of the two cell lines were fixed and processed for double immunofluorescence using the rabbit anti-MarvelD3 antibody and a mouse monoclonal antibody against occludin. The samples were first analyzed by epifluorescence microscopy.

**Figure 4:**
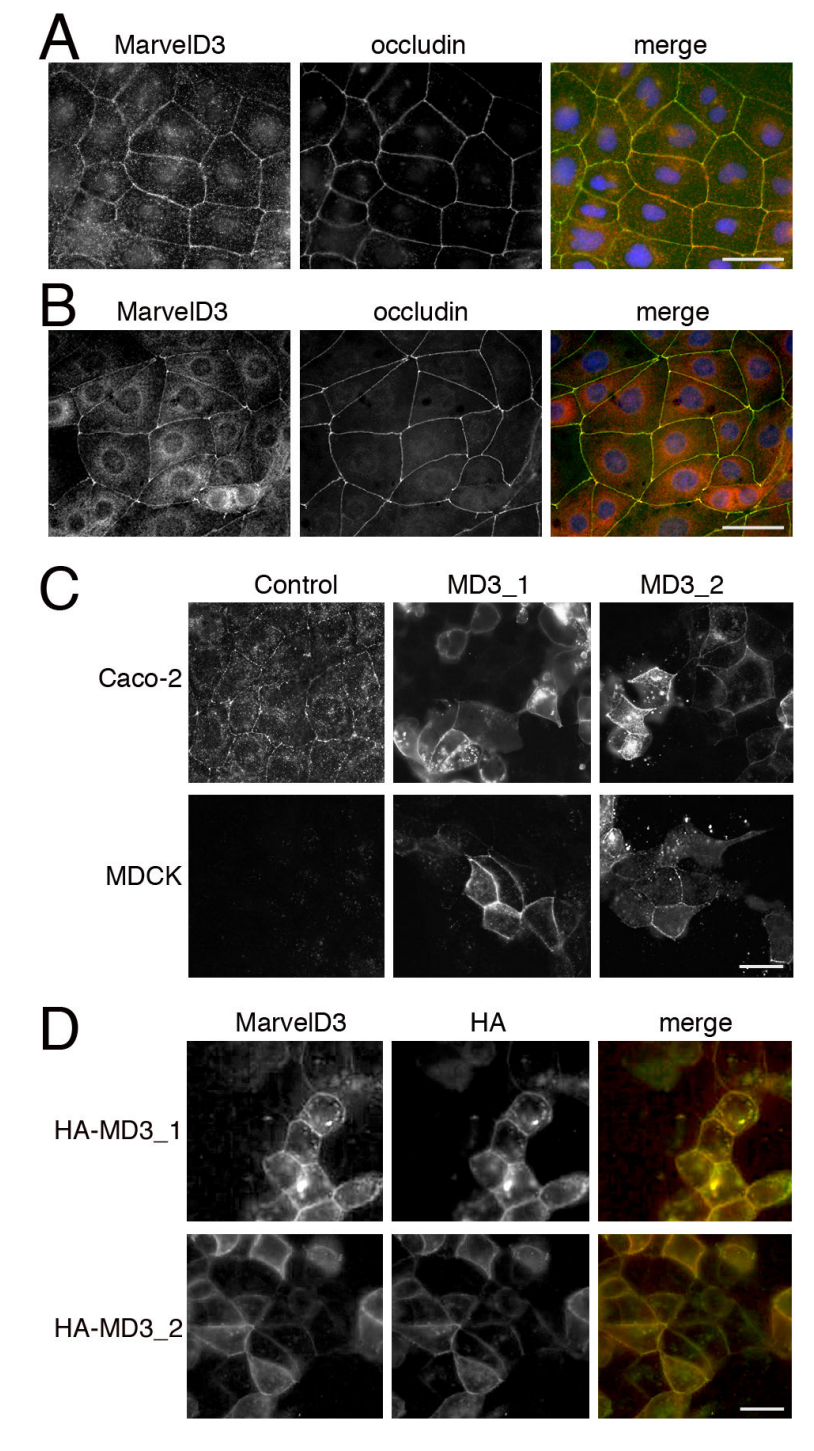
**Localisation of MarvelD3 by epifluorescence microscopy**. (A, B) Immunostaining of Caco-2 (A) and HCE (B) cells with anti-MarvelD3 and anti-occludin antibodies. Cells were cultured on glass coverslips and fixed with methanol. (C) Localisation of MarvelD3 constructs in Caco-2 and MDCK cells. Caco-2 and MDCK cells were transfected with full length constructs of MarvelD3 isoform 1 (MD3_1) and isoform 2 (MD3_2). The samples were then fixed with methanol and labelled with the anti-MarvelD3 antibody. Note, both isoforms are equally distributed and localise to cell junctions. (D) Expression of HA-tagged MarvelD3. Caco-2 cells were transfected with HA-tagged variants of the MarvelD3 isoforms and then double labelled with anti-HA and anti-MarvelD3 antibodies. Bars, 10 μm.

Fig. 4 shows that the anti-MarvelD3 antibody stained cell-cell contacts in Caco-2 (Fig. 4A) and HCE (Fig. 4B) cells. There was also some cytoplasmic and nuclear staining. However, the junctional staining was specific as it disappeared when MarvelD3 was depleted by RNA interference (see below). In both cell lines, MarvelD3 and occludin co-localised, suggesting that MarvelD3 is a component of the apical junctional complex.

We next transfected cDNAs encoding the two isoforms into Caco-2 and MDCK cells to confirm the localisation observed for the endogenous protein. Fig. 4C shows that both isoforms were enriched at cell-cell contacts, supporting the staining observed for endogenous protein in panel A. Control MDCK cells did not reveal any staining for MarvelD3, possibly due to the species difference as the antibody was made against a sequence of the human protein that shows little conservation in the canine protein. Expression of N-terminally HA-tagged MarvelD3 isoforms in Caco-2 cells also resulted in staining of cell-cell contacts with anti-HA and anti-MarvelD3 antibodies (Fig. 4D). These data indicate that both isoforms of MarvelD3 localise to cell-cell contacts in epithelial cells.

**Figure 5:**
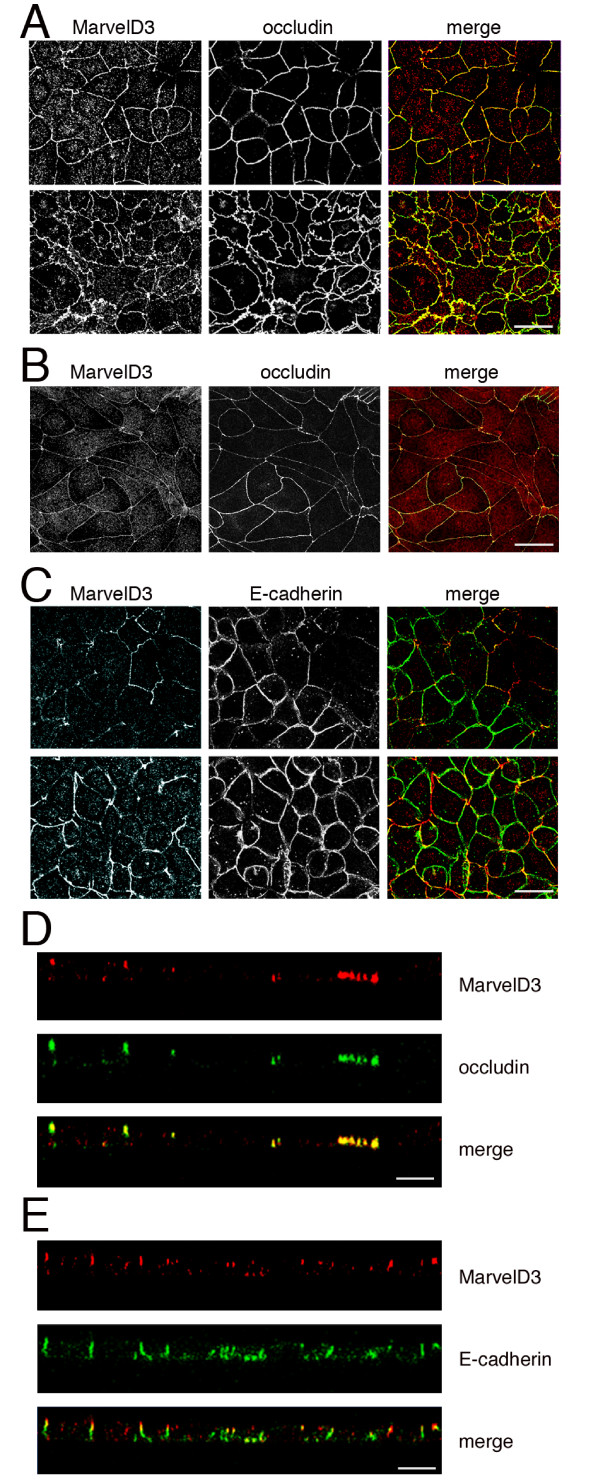
**Localisation of MarvelD3 by confocal microscopy**. Caco-2 cells grown on filters (A, C, D, E) and HCE (B) cells grown on coverslips were fixed and processed for immunofluorescence with the indicated antibodies. The samples were then analyzed by confocal microscopy. Panels A, B, and C are xy sections, and D and E are reconstitutions of serial z line scans. In panels A and C, two sections from different samples are shown that were both taken at the interface between the tight and adherens junctions to facilitate comparison between the two different labels in each specimen. Note, occludin and MarvelD3 tightly follow each other in and out of the focal plane. Bars, 10 μm.

We next used confocal microscopy to analyse the expression of MarvelD3 in more detail. Fig. 5A and 5B show that MarvelD3 and occludin co-localised at cell junctions in Caco-2 (Fig. 5A) and HCE cells (Fig. 5B) and in the same focal plane (note that the staining patterns of the two proteins enter and leave the focal plane at the same sites). In contrast, MarvelD3 and the adherens junction marker E-cadherin localised in different focal planes (Fig. 5C). The concentration of MarvelD3 at the apical end of the lateral membrane together with occludin, apical to the lateral E-cadherin staining, was also observed in z-projections reconstituted from serial z line scans (Fig. 5D and 5E). These data thus demonstrate that MarvelD3 co-localises with occludin, but not E-cadherin, at the junctional complex, indicating specific association with tight junctions.

### Functional characterisation of MarvelD3 at the tight junction

**Figure 6:**
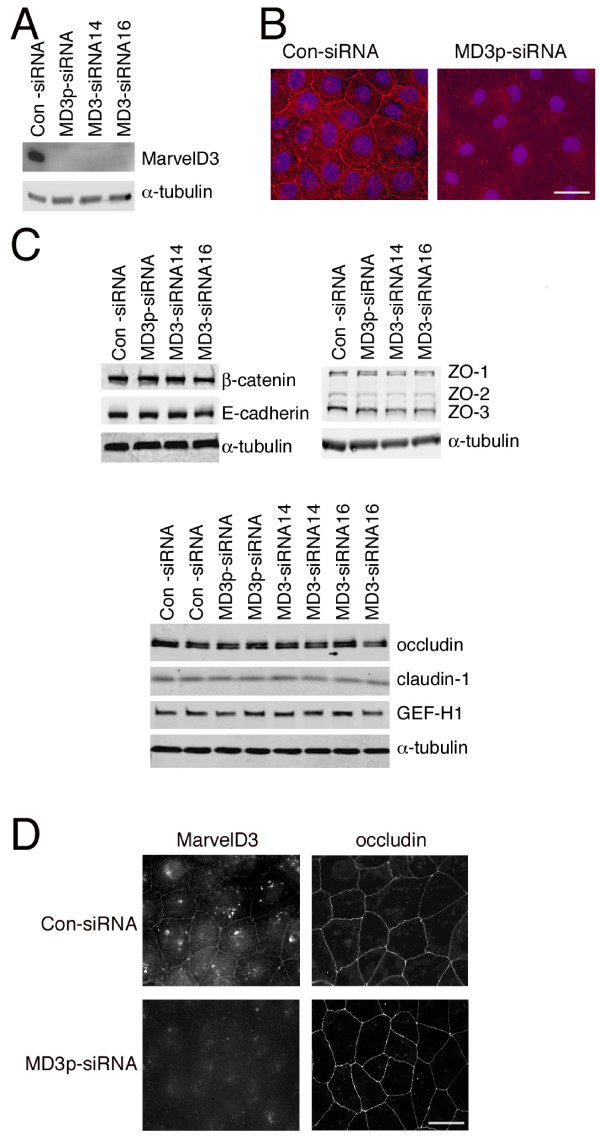
**Depletion of MarvelD3 in Caco-2 cells**. Caco-2 cells were transfected with the indicated siRNAs and then processed for immunoblotting (A and C) or immunofluorescence (B and D). (A, C) Cells were immunoblotted with antibodies against MarvelD3 and α-tubulin (A) or against a selection of tight and adherens junction proteins as indicated. Note, the expression levels of none of the junctional proteins apart from MarvelD3 were affected by depletion of the latter protein independent of whether filter- or glass-grown cells were analysed. Bar, 10 μm. (C) The lower panel in C shows duplicate cell extracts for each type of siRNA transfection. (B) Immunofluorescence staining of cells labelled with anti-MarvelD3 antibodies and Hoechst dye to stain DNA. Note, reduced levels of MarvelD3 were seen following transfection with MarvelD3 siRNAs. (D) siRNA transfected cells were labelled with anti-MavelD3 and anti-occludin antibodies. Bar, 10 μm. Note, knockdown of MarvelD3 did not appear to affect occludin distribution.

To begin to address a functional role of MarvelD3 at the tight junction, we used siRNA to deplete MarvelD3 and looked for the effects this had on the localisation and expression levels of other protein constituents of the apical junctional complex. Efficient knockdown of MarvelD3 was achieved with a pool of siRNAs as well as the two individual siRNAs identified above (Fig. 6A). Depletion of MarvelD3 on immunoblots was mirrored by the absence of junctional staining by immunofluorescence (Fig. 6B), indicating that knockdown of MarvelD3 was sufficient to efficiently deplete the junctional pools of MarvelD3.

We next tested whether depletion of MarvelD3 affected expression levels of other junctional proteins. Expression of the tight junction proteins occludin, claudin-1, GEF-H1, ZO-1, ZO-2 and ZO-3, and the adherens junction proteins E-cadherin and β-catenin remained unchanged (Fig. 6C). We were not able to detect tricellulin using an available commercial antibody. Similarly, immunofluorescence analysis did not reveal any effects of MarvelD3 depletion on the distribution of other junctional proteins (Fig. 6D: shown is occludin). Although we cannot exclude minor alterations based on these data, depletion of MarvelD3 does not seem to affect the overall distribution of major junctional components.

An important function of tight junctions is the generation of a tight seal between neighbouring cells of the monolayer, which restricts the movement of ions and solutes through the paracellular pathway [1]. To determine whether or not MarvelD3 plays a role in the assembly of the barrier or in the regulation of ion permeability, we compared the TER of monolayers formed by control Caco-2 cells with those formed by Caco-2 cells depleted of MarvelD3. To follow assembly and monolayer formation of the junction, cells were seeded first on plastic for transfection of the siRNA. 24 hours after transfection, the cells were re-plated onto permeable supports either in normal tissue culture medium (direct plating) or at low Ca^2+ ^concentrations, which are insufficient to support junction formation. Junction assembly was then initiated 24 hours after plating by switching the cells to normal Ca^2+ ^concentrations (Ca^2+ ^switch) [45], by which time depletion had already occurred. The monolayers were followed for a further 48 hours by measuring TER and then analysed for protein expression and paracellular tracer permeability.

**Figure 7:**
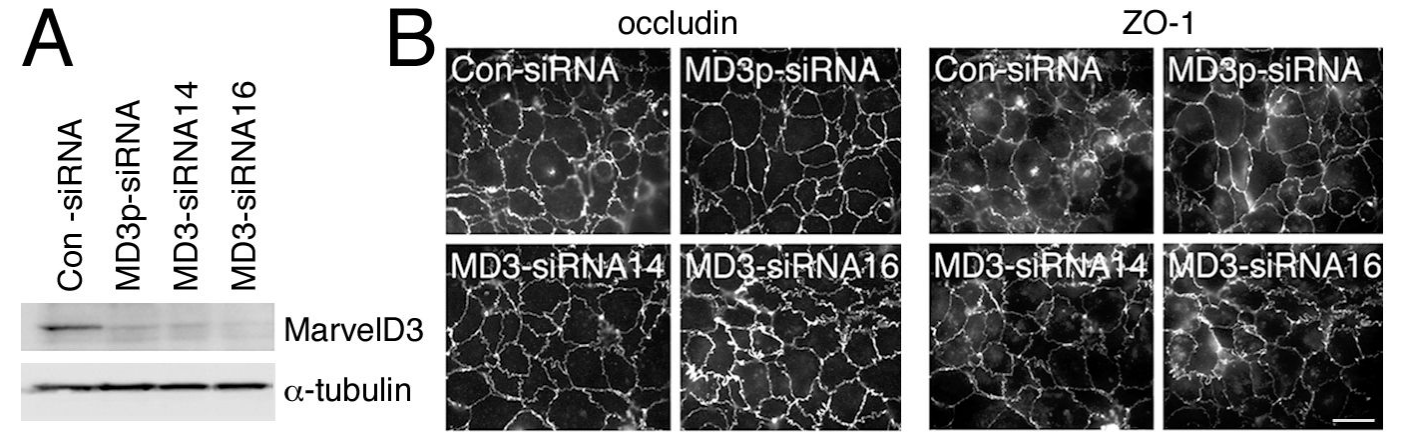
**Depletion of MarvelD3 and tight junction assembly**. (A) Control and siRNA-transfected Caco-2 cells were plated on filters one day after transfection. The cells were lysed three days later and expression of MarvelD3 and α-tubulin was determined by immunoblotting. (B) Cells treated as those in panel A were fixed and processed for immunofluorescence at the end of the incubation period. Shown are epifluorescence images of samples labelled for the tight junction markers occludin and ZO-1. Bar, 10 μm. Note, depletion of MarvelD3 did not affect monolayer integrity and appearance.

**Figure 8:**
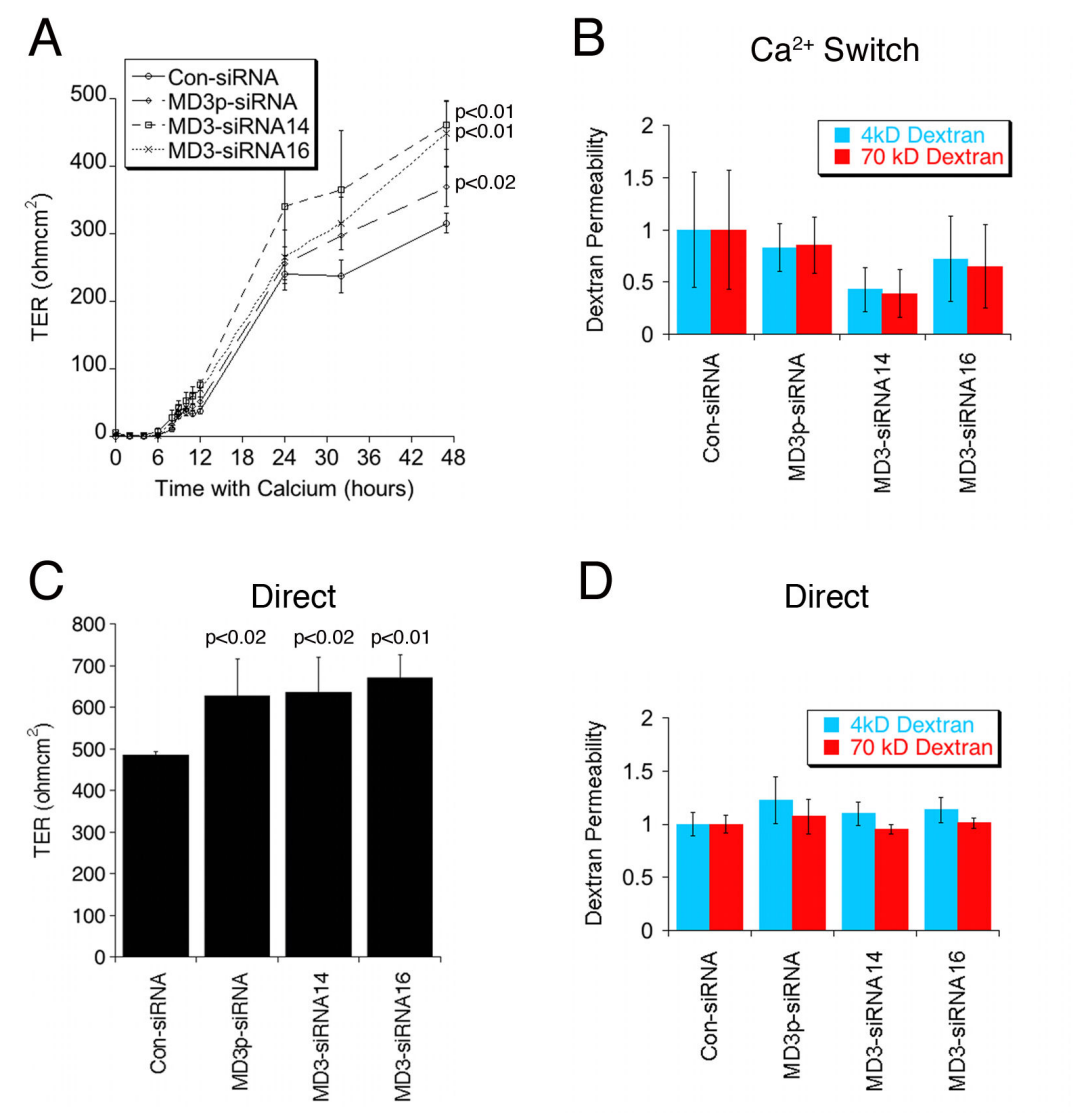
**Depletion of MarvelD3 and epithelial barrier properties**. Cells were cultured as in fig. 5 either using the Ca^2+ ^Switch (A, B) or Direct plating (C, D) protocol. TER and fluorescent dextran permeability using 4 kD and 70 kD dextran were measured as indicated. The amount of dextran diffused to the basolateral side of the monolayer was normalised against the average value obtained from control cells. Shown are averages ± 1SD of quadruplicate samples of a typical experiment. The indicated p values were obtained with a t-test comparing knockdown with control values; in panel A, the p values refer to the final TER values. Note, MarvelD3 knockdown had no significant effect on diffusion of either dextran tracer across monolayers in either the Ca^2+ ^switch experiment (B) or those plated directly into complete culture medium (D). The apparent decreases in the mean values in panel B obtained for single siRNA transfections were neither statistically significant nor did they reflect a trend observed in other experiments. However, final TER values were elevated in both culture conditions in all performed experiments.

Fig. 7A shows that MarvelD3 was still efficiently depleted at the end of the incubation period. Similarly, no effects on monolayer morphology and localisation of junctional proteins were observed (Fig. 7B: shown are occludin and ZO-1). TER measurements revealed that formation of functional tight junctions still occurred at similar kinetics as in control cells (Fig. 8A). However, MarvelD3 depleted cells reached higher resistance values. Determination of tracer permeability using 4 kD and 70 kD fluorescent dextrans did not suggest any defects in barrier formation or significant alterations in tracer diffusion (Fig. 8B). Cells that were directly plated in normal medium, and were hence 24 hours longer in normal Ca^2+ ^medium, also formed functional tight junctions and reached stable TER values by the end of the incubation time (Fig. 8C and 8D). As the Ca^2+^switch cells, directly plated MarvelD3 depleted cells had reached 25% higher electrical resistance values than control cells.

Taken together, these results indicate that MarvelD3 depletion does not affect the formation of functional tight junctions, but that MarvelD3 levels are a determinant of paracellular ion conductivity.

## Discussion

In this study, we have shown that MarvelD3, a four-pass transmembrane protein, co-localises with the tight junction protein occludin, apical to the adherens junction protein E-cadherin, indicating that MarvelD3 is a third tight junction-associated Marvel domain protein. A functional analysis using RNA interference-mediated depletion indicates that MarvelD3 is not essential for junction assembly and the formation of a functional paracellular diffusion barrier, but the observed increase in TER in depleted cells indicates that MarvelD3 is a determinant of paracellular ion permeability.

Our data demonstrate that MarvelD3 associates with the junctional complex of intestinal and corneal epithelial cells, and its expression was detected in multiple mouse tissues and cultured epithelial and endothelial cell lines. MarvelD3 thus seems to be a widely expressed protein and hence likely to be a component of tight junctions with different functional properties.

MarvelD3 is expressed as two isoforms and both were recruited to the cell periphery in transfected cells in a manner similar to the endogenous protein, suggesting that both isoforms associate with junctions. Our attempts to generate isoform-specific antibodies, which are required to study endogenously expressed isoforms at the protein level, have so far failed. Based on reverse transcription PCR results, however, both isoforms seem to be widely expressed. An aim of future studies will be to differentiate between MarvelD3 isoforms at the protein level and to determine whether there are any isoform-specific differences in expression, localisation and/or function, as has been suggested for occludin [46,47].

Occludin and tricellulin not only localise to tight junctions, but have been shown to associate with the junctional intramembrane strands observed in freeze fracture replicas [27,48]. It will thus be important to determine whether MarvelD3 also associates with these structures and whether the Marvel domain, which is found in all three proteins, is important for strand association [42]. The Marvel domain is found in proteins such as MAL that are thought to associate with cholesterol-rich microdomains in cell membranes [42]. As occludin has previously also been shown to associate with cholesterol-rich microdomains [49], it is possible that Marvel domain proteins affect tight junctions via such membrane microdomains. At present, however, the precise role of cholesterol in tight junctions is not clear [50-52].

Given the membrane morphology of the tight junction in which the outer leaflet of the plasma membrane becomes closely apposed to that of an adjacent cell, proteins with potential properties to influence close membrane/membrane contacts, such as Marvel domain proteins, may prove of significant interest in understanding the structure and function of tight junctions. Tricellulin has been suggested to play a structural role in tricellular corners, which would be compatible with a role in forming and/or stabilising membrane/membrane contacts [27,39]. Surprisingly, however, the analysis of patients carrying tricellulin mutations has so far only revealed defects in the cochlea and not other organs [38]. In the case of occludin, depletion and knockout experiments as well as expression studies with dominant mutations have thus far not suggested a role in tight junction structure [28,34,37,53]. However, it has recently been reported that depletion of occludin results in a more even distribution of tricellulin along the entire junctional length [41]. Hence, different Marvel domain proteins may be able to compensate for each other. With this in mind, it will be important to design approaches to study Marvel domain proteins as a whole and to elucidate a function for the Marvel domain itself.

Depletion of MarvelD3 in Caco-2 cells resulted in an increase in TER but did not affect permeability to fluorescent dextran tracers. Since occludin overexpression was previously observed to increase TER [28], we wondered whether the increased TER observed in the present study could be attributable to elevated levels of occludin expression. Immunoblotting cell lysates from MarvelD3 knockdown cells however showed occludin levels, as well as levels of ZO-1, ZO-2 and ZO-3, to be unchanged. Neither did we observe any significant changes in the distribution of these proteins by immunofluorescence. However, at this point it cannot be excluded that more subtle changes in occludin distribution might have caused the increase in TER. Occludin knockdown has been shown to reduce expression levels of claudins 1 and 7 and increase levels of claudins 3 and 4 in MDCK cells [37]. Given the importance of claudin family members for paracellular ion permeability [12-14], it is possible that MarvelD3 depletion affected claudin expression or function; though we have not been able to detect differences in claudin-1 expression. However, it is possible that other claudins are affected or that claudin activity is regulated by MarvelD3.

MarvelD3 shows distant structural similarity to occludin. Occludin and tricellulin are located in tandem on human chromosome 5 and it has been speculated that they may have arisen from gene duplication during phylogenetic evolution [27]. In contrast, MarvelD3 is located on chromosome 16 but is also expressed by all vertebrates, including birds, fish, amphibians and mammals. In contrast to occludin and tricellulin, all known isoforms and variants of MarvelD3 have short C-terminal cytoplasmic domains that are also distinct from each other. It is therefore unlikely that they are able to interact with ZO-1 as those of occludin and tricellulin do [38,43]. In contrast, the N-terminal domain of MarvelD3, which is shared by both isoforms, is long in comparison to that of occludin. Intriguingly, the N-terminal domain of MarvelD3 is not as well conserved as the rest of the protein; however, it contains regions that are better conserved than others, which might be of functional relevance. In the case of occludin, this domain is thought to have a regulatory function and interacts with a ubiquitin ligase [32,54]. As the N-terminal domains of tight junction-associated Marvel domain proteins are distinct in length and structure, they might provide structural links to different types of junctional regulatory mechanisms.

## Conclusions

Our experiments identify MarvelD3 as a novel member of the occludin family, a subgroup of the Marvel domain proteins. MarvelD3 is expressed as two isoforms that show a broad tissue distribution. Similarly to occludin, normal MarvelD3 expression is not essential for tight junction formation. Nevertheless, knockdown of MarvelD3 affects the paracellular barrier properties of tight junctions by mechanisms that still have to be identified. Tight junctions and occludin have not only been linked to epithelial barrier functions but also to the signalling mechanisms that guide epithelial proliferation and differentiation [33,55,56]. Hence future studies will not only have to address the precise roles of MarvelD3 and other family members in tight junction structure and permeability properties, but also in the regulation of the subcellular signalling mechanisms that guide epithelial proliferation, differentiation and tissue formation.

## Methods

### Cell culture

Caco-2, HCE, MDCK, HaCaT, HepG2 cells were grown in DMEM (Invitrogen, Paisley, UK) with 10% (HCE, MDCK, HaCaT, HepG2) or 20% (Caco-2) foetal bovine serum (Invitrogen, Paisley, UK) [7,57,58]. PNT-1a and PNT2-c2 were cultured in RPMI 1640 (Invitrogen, Paisley, UK) supplemented with glutamine and 10% foetal bovine serum, ARPE-19 cells in a 1:1 mixture of DMEM and Nutrient Mixture F12 containing 10% foetal bovine serum and L-glutamine, hCMEC/D3 in EGM-2 medium (Cambrex BioScience, Wokingham, UK), and HCEC-B4G12 in human Endothelial-SFM containing 10 ng/ml FGF-2 (Invitrogen, Paisley, UK). All cell lines were grown in the presence of penicillin (100 U/ml) and streptomycin (100 μg/ml; Invitrogen) at 37°C in a 5% CO_2 _atmosphere.

### Antibodies

A rabbit anti-MarvelD3 antibody was generated against a peptide with the sequence CAPDRGPRRDTHRDAG and was affinity purified using the same peptide coupled to epoxy-activated sepharose beads. Antibodies against the following proteins were as previously described or were from commercial sources: ZO-1, -2, -3 and GEF-H1 [59]; α-tubulin [60]; β-catenin (Sigma Aldrich and Santa Cruz Biotechnology); occludin, tricellulin and claudin-1 (Invitrogen); and E-cadherin (BD Biosciences); HA-epitope [61].

### Immunofluorescence

Caco-2 or HCE cells were grown on glass coverslips or filters, as specified, for at least 3 days and then processed for immunofluorescence as described [28,53]. Briefly, cells were either fixed directly in methanol or first extracted on ice with 0.1% Triton X-100 for 1 minute and then fixed in methanol at -20°C for 10 minutes. Cells were rehydrated in PBS for 5 minutes and blocked for 15 minutes with 0.5% BSA in PBS (PBS-BSA). Coverslips were incubated with primary antibodies for 2 hours at room temperature, washed three times with PBS-BSA and incubated with secondary antibody and Hoechst nuclear stain for 1 hour at room temperature. Filters were incubated in primary antibody overnight at room temperature in a moist atmosphere, washed three times with PBS-BSA and incubated in secondary antibody with Hoechst for 1.5 hours at room temperature. Coverslips and filters were then washed two times with PBS-BSA and once with PBS before being mounted onto glass microscope slides with ProLong Gold mounting medium (Invitrogen, Eugene, OR). Images were acquired using a Leica Image capture epifluorescent microscope and a Zeiss LSM510UV confocal microscope with 63× oil immersion lenses. Brightness and contrast were adjusted with Adobe Photoshop CS4 software.

### cDNAs

cDNAs coding for full length untagged MarvelD3 isoforms were generated by PCR from reverse transcribed Caco-2 total RNA and from an EST clone obtained form Geneservice Source BioScience plc. The PCR fragments were cloned into pcDNA4-TOmychisB expression vector either as untagged versions or with a N-terminal HA-tag. The resulting cDNA constructs were verified by sequencing. Plasmids were transfected into Caco-2 and MDCK cells using Lipofectamine 2000 (Invitrogen) and analysed after 48 hours.

### siRNAs

For knockdown studies, Caco-2 and HCE cells were transfected with individual or a pool of siRNAs (MD3p-siRNA). The following MarvelD3-specific siRNAs were used (5' to 3' direction): siRNA13, GCGCAGGTGAACACGGAGT; siRNA14, GAGAGGAGGTGGAATATTA; siRNA15, CCGGAGAGAGACCAGGAGA; siRNA16, GCCGCAGACCCGAAAGTGA. All four target sequences are found in the N-terminus common to both isoforms of MarvelD3 (Thermo Scientific, Dharmacon). Used were On-Target plus siRNAs along with control On-Target plus siRNAs from the same provider. For transfections, Interferin transfection reagent (Polyplus-transfection Inc.) was used according to the manufacturer's instructions. Expression was then analysed between two and four days after transfection. Efficient knockdown was achieved with a total final siRNA concentration of 100 nM.

### Immunoblotting

Whole cell lysates were collected by adding SDS sample buffer (3% SDS, 15% glycerol, 0.0015% bromophenol blue, 0.25 M Tris HCl containing 150 mM DTT and 6 M Urea) to samples pre-washed once in PBS. The samples were incubated for 30 minutes at room temperature and were then homogenised through a 25G needle before analysis by SDS-PAGE. MarvelD3 protein levels were detected with a horseradish peroxidase conjugated donkey anti-rabbit antibody and enhanced chemiluminescence detection system (ECL, Amersham, Corp. Arlington Heights, IL). All other protein levels were detected using an Odyssey detector and IRDye-680- and IRDye-800CW-conjugated donkey anti-rabbit and donkey anti-mouse secondary antibodies (LI-COR).

### Reverse transcription PCR

Total RNA was isolated from tissues and cultured cells using standard procedures [62,63]. Reverse transcription was performed at 45°C using AMV reverse transcriptase (Promega Corp, Madison, Wisconsin, USA) for 1 hour. A sample of HUVEC cDNA generated from total RNA was kindly provided by Jay Stone (UCL Institute of Ophthalmology, London, UK). The indicated cDNAs were then amplified by PCR using FastStart Taq polymerase (Roche Diagnostics Ltd.) and an annealing temperature of 63°C. The following primers were used (5' to 3' direction): human MarvelD3 AAAAATCTAGATCAAAGAG TTCCAGACCACAG and AAAAATCTAGATTAAAATTCAAACATTTCTGCTGG for reverse transcription, and AAAAAGCTAGCAAATACTTGTGCACTGGGAGAG, AAAAAGCTAGCTC GGTAGCTACGCAGGGC, AAAAAGCTAGCTCGGTAGCCCTTTATGGCCA for PCR amplification; mouse MarvelD3 CACAAATGCAGATACTTGTGCACAGG, CTAAAACTCAAGCATTTCTGTGGGC, TTAGAGCGTCCCGGACCACAGGTA; GAPDH (human and mouse) ATCACTGCCACCCAGAAGAC and ATGAGGTCCACCACCCTGTT.

### Paracellular permeability and TER measurements

Caco-2 cells were transfected with control, pool or individual siRNAs against MarvelD3 in six well plates. The day after transfection, cells were trypsinised and plated at confluent density on Transwell filters (Corning Inc., Corning, NY) in quadruplicates, either in low calcium medium, for Ca^2+ ^switch, or directly into complete DMEM. After 24 hours, the medium was changed for fresh complete DMEM and cells were incubated at 37°C for 10 minutes before starting to measure TER. TER was recorded every 2 hours for the first 12 hours, then at 24, 36 and 48 hours by applying an AC square wave current of ± 20 μA at 12.5 Hz with a silver electrode and measuring the voltage deflection elicited with a silver/silver-chloride electrode using an EVOM (World Precision Instruments), as previously described [28]. To measure paracellular flux, 1 hours before the experiment, medium was replaced with 1000 μl complete DMEM on the basolateral side and 250 μl on the apical side and the cells returned to 37°C. Tracer solution containing 4 kD FITC-dextran and 70 kD Rhodamine-B-dextran was added to the apical side and incubated at 37°C for 4 hours [64]. 200 μl samples were collected from the basolateral side and fluorescence was determined using a microplate reader. Fluorescence of the apical solutions was used to determine total values.

## List of abbreviations

TER: transepithelial electrical resistance; Marvel domain: MAL and related proteins for vesicle traffic and membrane link domain.

## Authors' contributions

All four authors performed experiments. ES, MSB and KM planned the project, designed experiments and wrote the paper. All authors read and approved the final manuscript.
